# Application of Bioluminescence Imaging for *In Vivo* Monitoring of Fungal Infections

**DOI:** 10.1155/2012/956794

**Published:** 2011-10-27

**Authors:** Matthias Brock

**Affiliations:** Microbial Biochemistry and Physiology, Leibniz Institute for Natural Product Research and Infection Biology, Hans Knoell Institute, Beutenbergstr. 11a, 07745 Jena, Germany

## Abstract

Fungi can cause severe invasive infections especially in the immunocompromised host. Patient populations at risk are increasing due to ongoing developments in cancer treatment and transplantation medicine. Only limited diagnostic tools and few antifungals are available, rendering a significant number of invasive fungal infections life threatening. To reduce mortality rates, a better understanding of the infection processes is urgently required. Bioluminescence imaging (BLI) is a powerful tool for such purposes, since it allows visualisation of temporal and spatial progression of infections in real time. BLI has been successfully used to monitor infections caused by various microorganisms, in particular bacteria. However, first studies have also been performed on the fungi *Candida albicans* and *Aspergillus fumigatus*. Although BLI was, in principle, suitable to study the infection process, some limitations remained. Here, different luciferase systems are introduced, and current approaches are summarised. Finally, suggestions for further improvements of BLI to monitor fungal infections are provided.

## 1. Introduction

Bioluminescence imaging (BLI) is a noninvasive technique that can be used to track microorganisms in living animals. To use this technique cells are generated that emit light from enzyme-catalysed oxidation reactions. Light emission is detected by highly sensitive charged coupled device (CCD) cameras that allow the collection of single photons [1]. The light-generating enzymes are generally called luciferases, although several different luciferases from a wide variety of organisms and without structural relatedness are known [2]. Nevertheless, all characterised luciferases have in common that they need oxygen for the light-emitting reaction but use different substrates and cofactors and emit light at different wavelengths. A short summary of key features of some frequently used luciferases that will be described in more detail is shown in Table 1.

BLI has been used to monitor gene expression and disease progression caused by pathogenic bacteria [3]. Furthermore, the system has also been applied to monitor growth of implanted tumour cells for investigating the effectiveness of therapeutic approaches [4, 5]. In terms of eukaryotic microorganisms such as fungi, bioluminescence imaging has mainly been used for highly sensitive monitoring of gene expression [6, 7], whereas infection studies on fungi are basically limited to the dimorphic yeast *Candida albicans* [8, 9] and the filamentous ascomycete *Aspergillus fumigatus *[10, 11]. These investigations have shown that BLI can be used as a powerful tool to study the infection process, but a fine-tuning of the system is required to increase its suitability. Thus, this paper summarises the key features of different luciferase systems, reports on the results obtained for fungal infections, and provides suggestions for future applications and improvements.

## 2. Bacterial Luciferases

For the generation of bioluminescent bacteria generally a *lux* operon from a prokaryotic origin [12] is used. This operon not only harbours genes coding for the heterodimeric luciferase (*luxA* and *B*) with a native mass of approximately 77 kDa [13] but also genes required for the production of the luciferase substrate (*luxC*,* D*, *E*). The latter genes are responsible for production of a long-chain aldehyde that is oxidised together with a reduced riboflavin phosphate (FMNH_2_) for the light-generating reaction [14]. To enable a heterologous expression of the *lux* operon in various bacteria, several optimisation strategies have been applied involving the use of different promoters and codon adaptations as required by the respective host [15]. Such adaptations allowed studying the infection process of several bacteria such as *Salmonella typhimurium*, *Listeria monocytogenes*, *Bacillus anthracis, Mycobacterium tuberculosis*, and others [3, 16–19].

Besides using the *lux* operon for virulence studies, it is also used as a reporter system to study promoter activity in bacteria under various environmental conditions. Here, although the reporter system is functional when all genes are located in a single operon, some substrate limitations can occur that limit the linearity of the reporter system. Recent studies have shown that this limitation is circumvented by independent and constitutive expression of the substrate-producing genes *luxC*, *D*, *E* and by only controlling expression of the luciferase-encoding genes by the promoter of interest [20].

Attempts have also been made to use the bacterial system for expression in eukaryotes since the bacterial *lux* operon provides the only system for which the genes for substrate synthesis are known. This allows the production of autobioluminescent cells without the need for external addition of luciferase substrates. However, since eukaryotes need a promoter controlling the expression of each single gene and, additionally, require a codon adaptation of the *lux* genes, studies using *lux* genes in eukaryotes are rather limited. Recently, it was possible to transfect mammalian HEK293 cells with a *lux* cassette harbouring codon-optimised genes linked by internal ribosomal entry sites to limit the number of promoter elements due to bicistronic gene expressions [21]. By this method it was possible to detect a minimum of 20,000 HEK cells *in vitro*. Additionally, after subcutaneous injection it was possible to redetect these *lux* cassette-expressing cells from living mice [21]. However, although this system allows the continuous long-term observation of a cell population without the need for external substrate addition, the system still possesses several drawbacks. Despite the constitutive expression of a flavin reductase gene, the amount of reduced FMNH_2_ appeared limited in the eukaryotic system since the addition of FMNH_2_ to the culture medium strongly increased luminescence signal intensity. Thus, although the substrate for luciferase can be autonomously produced by the cell, one of the essential cosubstrates remains limited. Furthermore, the use of these expression cassettes in other eukaryotes such as fungi will need species-specific adaptations involving time-consuming cloning procedures. Additionally it should be noted that, despite synthetic optimisation of *lux* genes for increased expression and production rates in HEK cells, luminescence signal intensity appeared rather low. Thus, combined with the short wave length of light emission (peak emission at 490 nm), this system may have limitations in monitoring cell proliferation from deeper tissues.

## 3. Beetle Luciferases

Studies using luciferases in research on eukaryotes frequently use enzymes that derive from luminous beetles (*Lampyridae*) or click beetles (*Elateridae*). The most prominent examples are the firefly luciferase from *Photinus pyralis* and the luciferase from the click beetle *Pyrophorus plagiophtalamus* [23]. Luciferases from these beetles are monomeric enzymes with a molecular mass of approximately 62 kDa and a protein sequence identity of around 50%. Additionally, the native protein sequences from both species display a C-terminal peroxisomal import sequence (SKL) for targeting to specialised peroxisomes of photocytes in the beetle lantern organ. Due to this targeting sequence, luciferases are also transported to peroxisomes of other eukaryotes as shown for mammalian cells, yeast, and plants [24, 25]. Although fireflies and click beetles are only distantly related, luciferases from both families use the same substrate, which is a heterocyclic water soluble acid (benzothiazoyl-thiazole) generally called luciferin. Luciferin is oxidised in an ATP-dependent manner leading to oxyluciferin, AMP, CO_2_, and light. The quantum yield of this reaction was determined as 0.88, which is the highest yield for any luciferase-catalysed reaction characterised so far [23]. Click beetle luciferases differ in the wavelength of emitted light ranging from green (548 nm) to orange (594 nm), and this range has been observed from individual beetles implying that different luciferases may be produced within the same insect [26]. In contrast, the firefly *P. pyralis* only produces a single luciferase with a peak intensity of light emission at 561–578 nm [22, 23]. However, it should be noted that a spectral shift to >610 nm (612–617 nm) occurs at pH below 7.5, temperatures above 35°C, addition of denaturants or several heavy metals [22, 27]. Thus, it has been shown that light produced by a firefly luciferase within a mammalian system (body temperature *≈*37°C) is red shifted [22]. Although this shift is accompanied by a reduced quantum yield [23], it is compensated by an increase in luciferase activity [22] and, thus, allows a better detection of microorganisms from deep tissues due to reduced light absorption from haemoglobin [28]. 

Genes for synthesis of luciferin are still unknown, and the external addition of this luciferase substrate is required to obtain bioluminescent signals. To investigate whether this requirement for luciferin supplementation limits the suitability of the system *in vivo*, the distribution of luciferin was studied in a murine system. In a qualitative analysis it was shown that 20 min after intraperitoneal injection luciferin was detectable in tissue homogenates from brain, skin, and liver. Furthermore, the repeated injection of 126 mg/kg of luciferin appeared to have no adverse toxic effect on animals [29]. However, quantitative analyses using either radioiodine labelled [30] or ^14^C-labelled luciferin [31] showed that distribution velocity and tissue accumulation vary depending on the route of luciferin application (intravenous versus intraperitoneal) and time of measurement after application. Both studies independently showed that luciferin availability especially in brain, muscles, and bones remains at low rates regardless of the application route and may, thus, act as a limiting factor for luciferase activity detection. Nevertheless, although substrate concentrations may be too low to allow substrate saturation for the light-emitting reaction, at least a qualitative assessment of bioluminescence can be envisaged from all targeted organs. Thus, although the number of injections and amount of luciferin applied to individual animals should be kept to a minimum, tracking of an infection at various time points and from different body sites should be possible despite the uneven substrate distribution in living animals.

As mentioned above, beetle luciferases require ATP for substrate oxidation, and this feature can be used to determine minute amounts of ATP released from cell lysates [32]. However, this feature of beetle luciferases may also act as a drawback during *in vivo* measurements. Due to the ATP dependence of these luciferases, not only the substrate luciferin may be limited, but also the ATP concentration, which is dependent on the physiological state of the cell. Thus, cosubstrate requirements, ATP in the beetle luciferase, and FMNH_2_ in the bacterial *lux* system are major disadvantages of these bioluminescence-based reporter systems.

## 4. Coelenterazine-Dependent Luciferases: Rluc and Gluc

Another type of luciferases frequently used as a reporter in eukaryotic cells oxidises the substrate benzylimidazo-pyrazinone coelenterazine (short coelenterazine) independent of ATP or FMNH_2_. Thus, these luciferases do not require a special physiological state of the host cell (despite the presence of oxygen) to act as a reporter and can be measured in cell-free extracts by coelenterazine addition. Two coelenterazine-dependent luciferases with different characteristics are mainly used as reporters: (i) the luciferase from the sea pansy *Renilla reniformis* (Rluc) and (ii) from the copepod *Gaussia princeps* (Gluc).

*Renilla* luciferase has been purified to homogeneity and biochemically characterized approximately 35 years ago. The enzyme is a monomer of 35 kDa that emits light at 480 nm. Although it is rather stable at elevated temperatures, it tends to irreversibly form inactive dimers and multimers when stored at concentrations exceeding 0.5 mg/mL [33]. Compared to the quantum yield of firefly luciferases, the quantum yield of Rluc with coelenterazine is approximately 10 times lower with only about 6-7%. Furthermore, also the substrate turnover rate appears rather low [33], which is a drawback in terms of sensitivity of the system. In addition, although the light emission at 480 nm is ideally suited to excite the *R. reniformis* green fluorescent protein, light at this wavelength is strongly absorbed by haemoglobin. Nevertheless, since bioluminescence background signals from animals are generally very low, Rluc has been successfully used to generate reporter cells for *in vivo* monitoring of cancer development in murine systems [34–36]. However, it has been reported that the multidrug resistance MDR1 P-glycoprotein (Pgp) efficiently removes coelenterazine from the cell and may, thus, lead to an underestimation of bioluminescence by the Rluc system [37]. In contrast, such a substrate export has not been described for the luciferin of firefly luciferase. It has also been shown that the distribution of coelenterazine is strongly dependent on the route of application. Studies on Rluc-expressing *Trypanosoma brucei* cells showed that the parasites were detected at different body sites depending on coelenterazine injection either via the intravenous or the intraperitoneal route [38]. Thus, although firefly luciferin is also not evenly distributed between different body sites bioavailability appears superior compared to coelenterazine. In addition, coelenterazine tends to autooxidation in the presence of serum albumin which enhances background signals, a phenomenon that is not observed with firefly luciferin [39].

The luciferase from *Gaussia princeps* has several different features compared to Rluc. Gluc is naturally secreted [40] and assumed to keep predators' attention while the copepod escapes. The secretion signal of this luciferase is also active in other eukaryotic cells as exemplified by expression studies in CHO and HepG2 cells [41]. Although, as described for Rluc, native Gluc consists of a monomeric structure, its molecular mass of 19 kDa is significantly smaller than that of Rluc [42]. Furthermore, although coelenterazine is also used as a substrate and light at a wavelength of 480 nm is emitted in an ATP-independent manner, light intensity is much higher than that of Rluc [43]. The enzyme additionally possesses a very high thermostability retaining more than 50% activity after 30 min incubation at 95°C [44].

The secretion of Gluc has several advantages that may also turn into disadvantages of the system. Due to its high stability, Gluc can be easily identified from culture supernatants, thus enabling to detect intracellular events from the environment [43]. Additionally, the extracellular localisation of Gluc provides an easier access to the substrate coelenterazine that can be limited in Pgp-positive cells by using the intracellular Rluc system. However, the secretion of Gluc also leads to an uncontrolled distribution of Gluc via the blood stream causing elevated background levels and less distinct signals at the site where luciferase-producing cells are located. To minimise the drawbacks of secretion attempts have been made to produce membrane-bound Gluc by fusing the enzyme with the CD8 transmembrane domain and expressing the construct in T cells. This procedure resulted in increased signal intensities of transfected cells and allowed *in vivo* monitoring of T-cell recruitment to implanted cancer cells [45]. Thus, future experiments may favour specific Gluc versions rather than Rluc for *in vivo* imaging.

## 5. Luciferases as Reporter Systems in Fungi

First successful approaches to produce firefly luciferase in the yeast *Saccharomyces cerevisiae* were published in 1988 [46]. The native cDNA was cloned under control of the alcohol dehydrogenase promoter and allowed a maximum luciferase yield of 10 ng/mL of culture. However, this amount was assumed to be too low for use as a suitable *in vivo* reporter. Thus, different promoters were selected to increase luciferase production rates. Accompanied with optimised culture and assay conditions it was possible to detect luciferase activity from a minimum of 1 × 10^6^ intact yeast cells [47]. However, this sensitivity still appeared too low for using the firefly luciferase system as a suitable reporter for gene expression analyses from intact cells. A search for the problems causing this low sensitivity led to the speculation that the peroxisomal localisation of native firefly luciferase mediated by the C-terminal SKL sequence might limit the availability of substrates and thus causing low light emission. Indeed, removal of the peroxisomal-targeting sequence enhanced light emission of transformed cells by two to three orders of magnitude. In addition, growth speed of cells harbouring the modified luciferase gene was significantly enhanced compared to cells expressing the native gene sequence, indicating that the peroxisomal localisation affected the natural physiology of yeast cells [6]. Subsequently, luciferase assay systems were developed for yeast cells that expressed both, firefly and *Renilla* luciferase at the same time allowing two study two independent cellular responses by BLI [48, 49].

Similar to *S. cerevisiae*, first approaches to use the firefly luciferase as a reporter in the dimorphic yeast *Candida albicans* were only partially successful [50]. Expressions of the firefly luciferase under control of the phase-specific *WH11* promoter yielded no detectable bioluminescence. However, this negative result was attributed to the presence of several leucine residues encoded by CUG triplets, which are translated into serine in *C. albicans* and might have led to a nonfunctional protein [51, 52]. Thus, to generate a bioluminescent reporter system suitable to study gene expression in *C. albicans* the *Renilla* luciferase was used, because its gene does not contain in frame CUG codons. Indeed, use of Rluc led to well-detectable bioluminescence and was suitable to study gene expression of various genes in both cell-free extracts and intact cells [51]. Subsequently, the Rluc system was also used to investigate the effectiveness of a tetracycline-regulated expression system in *C. albicans* [53]. Later, the firefly luciferase was revisited. By exchanging all CUG codons from a vector carrying the firefly luciferase gene designed for expression in mammalian cells it was possible to generate bioluminescent *C. albicans *cells. This firefly luciferase system was suitable for use as a selection marker in the transformation of clinical wild-type isolates without the need for other growth-disturbing markers that could affect cellular physiology [54]. However, although this system worked well for *in vitro* analyses, the system displayed some problems in infection studies as will be outlined below.

Bioluminescent reporters have also been generated for use in filamentous fungi. In a trial for studying the expression of a xylanase gene from *Aspergillus oryzae* in the close relative *Aspergillus nidulans *a luciferase reporter system was used. Unfortunately, no details on the source of luciferase had been provided, and it can only be speculated that a firefly luciferase was used. However, the observed bioluminescence correlated with the expected expression pattern of the xylanase gene and indicated that BLI should be functional also in filamentous fungi [55]. Subsequently, BLI was discovered for investigating circadian rhythms in the ascomycete *Neurospora crassa* by using a firefly luciferase gene with improved codons for expression in mammalian cells [56]. However, initial studies revealed that light emission from intact cells was rather low and efforts were made to increase signal intensity. Thus, a fully codon-optimised synthetic version of the luciferase gene was constructed that emitted high bioluminescence signals when fungal transformants were grown on media supplemented with luciferin. By this method it was possible to follow the circadian expression of clock-controlled genes over several days [7].

In summary, although studies using luciferase systems in fungi are still rather limited, this technique seems to provide a powerful tool to study gene expression from intact cells. Additionally, the use of dual reporter systems, such as a combination of Rluc and firefly luciferase, allows the independent monitoring of gene expression by BLI, because both luciferases are highly specific for their respective substrate. 

## 6. Bioluminescence Imaging in Fungal Virulence: *Candida albicans*

Investigations using BLI for monitoring fungal infections *in vivo* are basically limited to *C. albicans* and *Aspergillus fumigatus*. Despite the small number of investigations, the detailed investigations on system limitations may help to design reporter systems that could improve the BLI technique also for other fungi.

As mentioned above, Doyle et al. constructed a firefly luciferase for use in *C. albicans* in which all CUG codons of a luciferase gene adapted for expression in mammalian cells were replaced by CUU codons allowing correct translation of the gene into a functional protein [54]. This system was, in principle, suitable to follow the induction of gene expression as shown by the induced expression of the hyphal-specific gene *HWP1* under hyphae-inducing conditions. However, when light emission was studied from constitutively active promoters, it was noted that bioluminescence was significantly lower in hyphae than in yeast cells although cell-free extracts from both cell types revealed similar bioluminescence levels. Thus, it was concluded that the uptake of luciferin might be hampered by the reorganised cell wall in *C. albicans* hyphae [54]. However, the same group also investigated the suitability of the system for studying pathogenesis in mice by BLI [8]. In a vulvo-vaginal infection model the system was suitable to visualise the persistence of *C. albicans* within the vaginal lumen. To allow substrate saturation in these experiments luciferin was directly applied to the vaginal lumen and light emission correlated with the number of *C. albicans* cells. Furthermore, in vulvo-vaginal infected mice a topical treatment with miconazole revealed clearance of the infection as visualised by BLI. However, imaging of systemic candidiasis was hampered by bioluminescence intensities that were too dim to follow pathogenesis, and this was attributed to the formation of hyphae during tissue penetration of *C. albicans* cells.

Although the explanations provided by the author referring to a limited substrate uptake by hyphae sound valid, other reasons could also explain why monitoring of systemic infections failed. First, a much stronger bioluminescence is required when studying infections from deep tissues compared to more superficial infections. This is due to strong light absorption from haemoglobin, and, as a general rule, it can be assumed that light emission intensity is reduced by a factor of 10 by each cm of tissue depth [16]. Although the firefly luciferase gene was codon adapted in terms of the in frame CUG codons, an inspection of the codon adaptation index (CAI) against highly expressed *C. albicans* genes by using the dCAIoptimizer reveals a CAI of only 27%. Thus, translation of the luciferase-coding RNA might have been limited by a shortage of the respective tRNAs. Synthesis of a fully codon-adapted luciferase gene might, therefore, significantly increase protein production rates and the accompanied bioluminescence signal intensity.

Another limitation of the gene sequence utilised could have derived from the peroxisomal-targeting sequence of the luciferase used for constructing the bioluminescent *C. albicans* cells. As outlined above, studies on *S. cerevisiae* showed that light emission is strongly reduced when luciferase is targeted to yeast peroxisomes [6]. Thus, not only the structure of the cell wall, but also the amount of peroxisomes and the available ATP content within these compartments may vary in *C. albicans* hyphae and may limit substrate availability for the luciferase reaction. Thus, the simple removal of the peroxisomal-targeting sequence might have enhanced the suitability of the system for studying systemic infections. At the moment, studies are under way to confirm both hypotheses.

However, due to the described limitations of studying systemic *C. albicans* infection by use of the firefly luciferase, Enjalbert et al. investigated the suitability of a *Gaussia* luciferase for monitoring *C. albicans* infections [9]. Since Gluc is naturally secreted, it was assumed that cellular barriers would not hamper substrate availability. However, to avoid a systemic distribution of Gluc, the gene was fused with the sequence coding for the glycophosphoinositol-linked cell wall protein PGA59. This strategy was similar to that described for trapping Gluc on the surface of T cells [45]. *C. albicans* cells producing the cell-wall-anchored Gluc were compared to *C. albicans* cells expressing Rluc, and the highly superior luminescence intensity of the Gluc system was confirmed. Light emission was in a range that allowed visualisation from intact cells even by the naked eye. Additionally, it was shown that, when using as constitutive expression system, no significant differences were observed between yeast cells and hyphae [9]. This system was suitable to study the progression of infection after subcutaneous, cutaneous, and vaginal infections. In cutaneous infections, coelenterazine was directly added to the abraded skin area, whereas in subcutaneous infections coelenterazine was supplied subcutaneously and in vaginal infections by applying the substrate to the vaginal lumen. By these methods it was ensured that coelenterazine reached the site of infection in sufficient amounts and light intensities correlated well with the fungal burden.

When the cell-wall-bound Gluc system was used to study progression of systemic infections, no satisfying results were obtained. Under *in vivo* conditions the detected luminescence intensities did not exceed background levels although homogenized kidneys incubated with coelenterazine revealed luminescence signals [9]. Several reasons may account for this failure to monitor systemic infections. One major problem, as discussed by the authors, might have derived from the limited distribution of coelenterazine after intraperitoneal injection. This seems reasonable, since a similar observation was made for experiments performed with *T. brucei* in which intraperitoneal or intravenous injection of coelenterazine revealed different results for the localisation of parasites [38]. However, only the intraperitoneal coelenterazine injection had been utilised to study systemic *C. albicans* infections, and it cannot be excluded that an intravenous substrate injection might have been able to track the infection. However, in terms of investigating the dissemination to yet unknown body sites, the problems of substrate availability cannot be neglected. Another problem might have been derived from the light emission wavelength of the selected luciferase. Gluc emits light at a peak wavelength of 480 nm with no emission above 600 nm [43]. Since haemoglobin and tissue absorbance of light are much less pronounced at wavelengths above 600 nm [1], the strong absorption at 480 nm accompanied by an autooxidation of coelenterazine might have hampered the detection of *C. albicans* from deep tissues. Thus, coelenterazine-based systems might only become suitable reporters for systemic *C. albicans* infections when substrates are developed that allow enhanced tissue distributions and a red shift of light emission.

## 7. Bioluminescence Imaging in Fungal Virulence: *Aspergillus fumigatus*

BLI has also been investigated for its suitability to monitor progression of *A. fumigatus* infections from living mice. For this purpose, the firefly luciferase codon optimised for expression in mammalian cells but without a peroxisomal-targeting sequence had been used [10]. To obtain strains with a constitutive luciferase production expression of the firefly luciferase was controlled by the glyceraldehyde-3-phosphate dehydrogenase promoter. Indeed, light emission was easily detectable from intact cells by either using a microplate reader or other bioluminescence imaging devices. Since *A. fumigatus* is a filamentous fungus, this observation indicates that the cell wall of hyphae does not necessarily cause problems for intracellular luciferin availability. The system was also suitable to study the antimycotic efficiency of several drugs under *in vitro* conditions.

**Figure 1:**
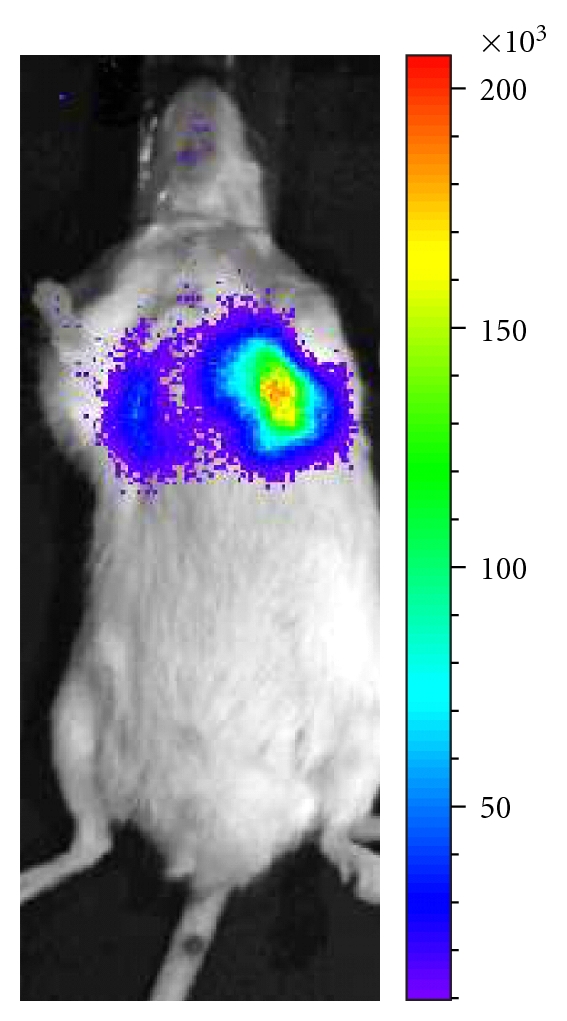
Bioluminescence imaging of invasive aspergillosis. The depicted mouse was immunosuppressed with cortisone acetate and infected intranasally with the bioluminescent *A. fumigatus* strain C3. Bioluminescence was acquired 28 h after infection. Light emission is detected from both lung lobes indicating the establishment of bronchopulmonary aspergillosis. (Figure kindly provided by O. Ibrahim-Granet).

When tested in a murine model for invasive bronchopulmonary aspergillosis (IBPA) by infecting mice immunocompromised by cortisone acetate, strong bioluminescence signals were obtained from the infected lung already 24 h after infection (Figure 1). Thus, this model provides the first example in which a deep tissue infection of a fungus was successfully monitored by BLI [10]. However, signal intensities strongly declined at later days, and histopathologic analyses showed a severe infiltration of immune effector cells consisting of mainly neutrophils that attacked fungal hyphae. Although subsequent analyses by qPCR showed that indeed the amount of living fungal cells did not increase three days after infection compared to a 24 h time point [11] the decline of the bioluminescence signal was surprising. Additionally, when an antibody-mediated neutrophil depletion strategy was used to render mice susceptible for IBPA, luminescence signals remained rather low and histopathology revealed a rapid and severe infiltration of monocytes to the site of infection.

To explain this phenomenon of decreasing luminescence signal intensity, it needs to be mentioned that all luciferases require dissolved oxygen for the oxidation of the respective substrate. Thus, it was speculated that the severe inflammatory process might cause anaerobic niches within the lung tissue [10]. In addition, within necrotic tissues the distribution of luciferin from the intraperitoneal site may be low causing substrate limitation at the site of infection. These speculations were supported by the fact that lungs removed at necropsy and injected with oxygen-saturated luciferin revealed luminescence signal intensities that correlated well with the expected fungal burden [10, 11].

The limitations of fungal quantification were less pronounced when mice were rendered leukopenic by using the cytostatic drug cyclophosphamide [11]. Under this regimen the amount of fungal biomass is heavily increasing, and no immune effector cells can be recruited to attack hyphae. Quantification of the bioluminescence signals showed that under this immunosuppression regimen signal intensity steadily increased until mice succumbed to infection. Thus, the firefly luciferase system, in principle, appears suitable to study the temporal and spatial progression of infection.

**Figure 2:**
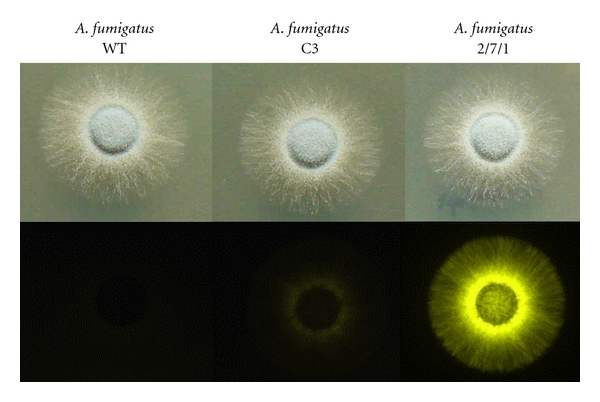
Visualisation of bioluminescence from *A. fumigatus* colonies. The *A. fumigatus* wild-type strain CBS144.89 and the bioluminescent strains C3 and 2/7/1 are shown. Strains were grown for 48 h in the presence of 1 mM D-luciferin on glucose minimal medium. The upper lane shows a daylight photograph, the bottom line bioluminescence images of the colonies acquired by a medium sensitive Versa Doc luminescence imaging system. Strain C3 carries four ectopic integrations of the *P. pyralis* luciferase gene codon adapted for expression in mammalian cells. Only a moderate light emission is detected. Strain 2/7/1 carries two integrations of the *P. pyralis* gene codon optimised for expression in *A. fumigatus*. Light emission is strongly enhanced.

Preliminary analyses have also shown that the bioluminescent *A. fumigatus* strain seems suitable to monitor progression of disseminated disease and signals were easily detected from all infected organs (unpublished results). However, to increase the sensitivity of the system, we are currently constructing and testing different *Aspergillus *species that carry a synthetic firefly luciferase gene adapted to the *A. fumigatus* codon usage (Figure 2). It is expected that the sensitivity from this construct will increase by at least a factor of 10. This increase would be sufficient to track even minute amounts of hyphae from infected tissues and should allow following the efficiency of antifungal therapy under *in vivo* conditions.

## 8. Conclusion

Different luciferase systems have been established for use in infection studies. The bacterial *lux* operon seems well applicable for studying bacterial infection, but appears less suitable for use in eukaryotic cells. Here, mainly coelenterazine-dependent and beetle luciferases are used, but both systems have limitations.

To overcome all problems the optimal bioluminescence reporter system should fulfil the following characteristics: (i) high expression rates, (ii) strong signal intensity with high quantum yield of the reaction, (iii) able to track deep infections with light emission far above 600 nm, (iv) independent from the host response (oxygen independent), (v) produce its own substrate intracellular, and (vi) should not affect physiology of the cell.

Unfortunately, at the moment such an optimal reporter system does not exist. Thus, the best suited system needs to be selected in regard to the scientific question. While superficial infections may be studied by coelenterazine-dependent luciferases, the limited substrate distribution and the short wavelength do not favour its selection for systemic infections. However, especially for gene expression studies in culture media, the Gluc system seems to provide an excellent candidate. Furthermore, besides coelenterazine and dissolved oxygen these luciferases are independent of additional cofactors that need to be provided by the host cell. Beetle luciferases are much larger proteins (three times the size of Gluc) and have been shown, under some circumstances, to affect fungal growth rate. Furthermore, they require cellular ATP as an essential cofactor that needs to be used from the cellular pool. However, light emission of these luciferases is above 600 nm under *in vivo* conditions, and luciferin distribution appears more homogenous. Thus, firefly luciferases may provide a better suited system for *in vivo* monitoring of fungal infections. Nevertheless, subcellular localisation and codon adaptations may play important roles for successfully applying this system to research.

In the future, investigations using BLI with other fungal pathogens will be performed. These studies will help to optimise luciferase reporter systems and will contribute to the understanding of pathogenicity mechanisms. A special feature will derive from the *in vivo* monitoring of the efficiency of antifungals in individual animals. Thereby, BLI will not only reduce the required number of animals but also allow identifying optimised treatment strategies to combat life-threatening fungal infections.

## Figures and Tables

**Table 1:** Key features of selected luciferases from different phylogenetic origins.

Luciferase origin	Organism (family)	Substrate	Cosubstrate	Composition (mass)	Localisation (native)	Peak emission (nm)
*Vibrio* spec.	Bacteria (*Vibrionaceae*)	Long-chain aliphatic aldehyde	O_2_; FMNH_2_	heterodimer (77 kDa)	Cytoplasm	490

*Photorhabdus* spec.	Bacteria (*Enterobacteriaceae*)	Long-chain aliphatic aldehyde	O_2_; FMNH_2_	heterodimer (77 kDa)	Cytoplasm	490

*Photinus pyralis*	Firefly (*Lampyridae*)	Benzothiazoyl-thiazole	O_2_; ATP	monomer (62 kDa)	Peroxisome	561–578*

*Pyrophorus plagiophthalamus*	Click beetle (*Elateridae*)	Benzothiazoyl-thiazole	O_2_; ATP	monomer (62 kDa)	Peroxisome	548–594

*Renilla reniformis*	Sea pansy (*Renillidae*)	Benzylimidazo-pyrazinone coelenterazine	O_2_	monomer (35 kDa)	Cytoplasm	480

*Gaussia princeps*	Copepod (*Metridinidae*)	Benzylimidazo-pyrazinone coelenterazine	O_2_	monomer (19 kDa)	Secreted	480

*Peak emission is temperature sensitive and gradually shifts to 612 nm at 37°C [22].
